# The atomic structure of human dystrophin spectrin-like repeat 24

**DOI:** 10.1107/S2053230X26003262

**Published:** 2026-04-23

**Authors:** Oakley Streeter, Ke Shi, Hannah Bui, Hideki Aihara, James M. Ervasti, Robert Evans, Joseph M. Muretta

**Affiliations:** a Department of Biochemistry, Molecular Biology, and Biophysics University of Minnesota, Twin Cities 6–155 Jackson Hall, 321 Church Street SE Minneapolis MN55455 USA; The Scripps Research Institute, USA

**Keywords:** dystrophin, spectrin-like repeat, Duchenne muscular dystrophy, cytoskeletal cross-linker

## Abstract

The structure of spectrin-like repeat 24 of human dystrophin was determined at 2.5 Å effective resolution. The structure exhibits a three-helix bundle fold, common to all spectrin-repeat family members, and shares a high degree of homology with existing structures of spectrin-like repeat 1 from dystrophin and utrophin. The structure provides molecular details of the atomic interactions that stabilize the repeat, including hydrophobic interactions and inter-helix and intra-helix salt bridges. *AlphaFold* models of the repeat are in excellent agreement with the structure, showing an all-atom r.m.s.d. of 1.13 Å. Accurate modeling of SR24 supports *AlphaFold* modeling of all 24 of the dystrophin spectrin-like repeats and the use of these models in predicting the molecular determinants of dystrophin stability, a key aspect of its biological function as a structural protein that cross-links actin filaments to the dystrophin–glycoprotein complex to mediate a mechanical connection between the cytoskeleton and the extracellular matrix.

## Abstract 2

The atomic structure of human dystrophin spectrin-like repeat 24 was determined at 2.14 Å resolution.

## 1. Introduction

Dystrophin is a cytoskeletal protein belonging to the spectrin superfamily and is responsible for linking the extracellular matrix (ECM)-binding dystrophin–glycoprotein complex (DGC) to the cortical actin cytoskeleton (Ervasti & Campbell, 1993 ▸; Rybakova *et al.*, 2000 ▸). Mutations in the dystrophin gene, *DMD*, cause Becker and Duchenne muscular dystrophy, in part by disrupting the mechanical linkage between actin and the DGC. Understanding the molecular determinants of dystrophin function is important for the development of dystrophin-replacement therapies to treat BMD and DMD, including gene therapies that deliver dystrophin surrogate-expressing expression cassettes to impacted tissues and exon-skipping therapies that cause the bypassing of exons that contain disease-causing mutations (Roberts *et al.*, 2023 ▸).

Dystrophin is composed of four major functional regions. The N-terminal actin-binding domain 1, composed of a tandem calponin homology domain, anchors the dystrophin N-terminal region to cortical actin filaments (Ervasti, 2007 ▸; Rybakova *et al.*, 1996 ▸, 2006 ▸). The central rod domain, composed of 24 tandem spectrin-like repeats interspersed with four flexible hinge-like sequences, has been hypothesized to provide mechanical stability to the actin–DGC linkage and contains binding sites for actin and other interaction partners. The C-terminal dystroglycan-binding domain anchors the protein to the DGC (Liu *et al.*, 2025 ▸; Wan *et al.*, 2025 ▸).

Each of the 24 ∼110-amino-acid spectrin-like repeats in the dystrophin rod domain is predicted to form three α-helices in a left-handed supercoil (Koenig *et al.*, 1988 ▸). The three-helix model is supported by existing structures of spectrin-like repeats from homologous proteins and a structure of the first spectrin repeat of dystrophin, SR1, the only previously published crystal structure of the first spectrin-like repeat in dystrophin (Muthu *et al.*, 2012 ▸). The role of spectrin-like repeats in dystrophin function remains poorly understood. The rod domain is broken up into discrete segments (SR1–SR3, SR4–SR19 and SR20–SR24) separated by flexible hinges (Koenig & Kunkel, 1990 ▸). Each segment is thought to resist mechanical deformation and thus to maintain separation of the hinge elements and to resist stretching forces. The spectrin-like repeats of dystrophin also act as binding sites for other interaction partners, including actin (SR11–S17), syntrophin (SR17 and SR22) and nNOS, which is anchored to SR16–SR7 via binding to syntrophin (Adams *et al.*, 2018 ▸; Amann *et al.*, 1998 ▸; Djinovic-Carugo *et al.*, 2002 ▸; Lai *et al.*, 2013 ▸; Rybakova *et al.*, 2002 ▸). Finally, the spectrin-like repeats of dystrophin are hypothesized to act as mechanical shock absorbers that unfold when extended beyond a critical unfolding force to protect the sarcolemma from mechanical shock (Bhasin *et al.*, 2005 ▸; Ervasti, 2007 ▸; Hua *et al.*, 2024 ▸; Le *et al.*, 2018 ▸).

The structure of spectrin-like repeat 24 (SR24) is of particular interest due to its presence in all dystrophin-replacement gene therapies and exon-skipping scenarios currently being used or tested in clinical trials (Roberts *et al.*, 2023 ▸). Furthermore, thermal stability measurements of dystrophin fragments show that portions of the protein containing SR24 exhibit higher thermal stability and resistance to urea denaturation than N-terminal portions of the protein (Legardinier *et al.*, 2008 ▸; Mirza *et al.*, 2010 ▸; Henderson *et al.*, 2011 ▸) and, as such, are hypothesized to help stabilize dystrophin as a whole. To gain insight into the molecular basis for the stability of SR24 and its potential structure in dystrophin-replacement therapies, we determined its three-dimensional structure using X-ray diffraction.

## 2. Methods

### 2.1. Cloning

The SR24 expression plasmid was constructed by Gibson assembly of a synthetic Gene Block (Gibson *et al.*, 2009 ▸). The DNA sequence for dystrophin residues 2933–3046 corresponding to accession No. NM_004006.2 was synthesized by Integrated DNA Technologies with 20 bp 5′ and 3′ extensions homologous to the expression plasmid, pTD68, a pET plasmid containing an N-terminal 6×His-SUMO tag followed by a multiple cloning site and a T7 terminator sequence (Aird *et al.*, 2018 ▸). This sequence spans the SR24 domain annotated in UniProt reference P11532 and includes two amino-acid residues prior to the dystrophin SR24 domain (2933–2934), SR24 (2935–3040) and six residues following SR24 that are present in the Hinge 4 region of the protein. The expressed residues were informed by *AlphaFold* predictions of dystrophin, which suggest that starting the expression construct at residue 2935 could potentially destabilize the first turn of the the spectrin-like repeat helix A and that the six residues following residue 3040 form a stable helix that is contiguous with SR24 helix C. The parent vector was engineered with AgeI and XhoI cut sites between the BamHI site 3′ to the SUMO tag followed by the T7 terminator and linearized by restriction digestion using AgeI and XhoI (New England Biolabs) followed by insertion of the Gene Block using Gibson Assembly Master Mix (New England Biolabs). The inclusion of the AgeI cut site in the expression plasmid resulted in three additional amino acids (STG residues 1–3 in the crystal structure) at the N-terminus of the crystallized protein. The assembled plasmid was transformed into competent *Escherichia coli* DH5α cells and plated onto 100 µg ml^−1^ ampicillin Luria–Bertani (LB) agar plates. Plasmids were purified from positive transformants and then sequence-verified by Sanger sequencing. Additional plasmid and recombinant protein reading frame details are provided in Table 1 ▸.

### 2.2. Protein expression and purification

**Figure 1:**
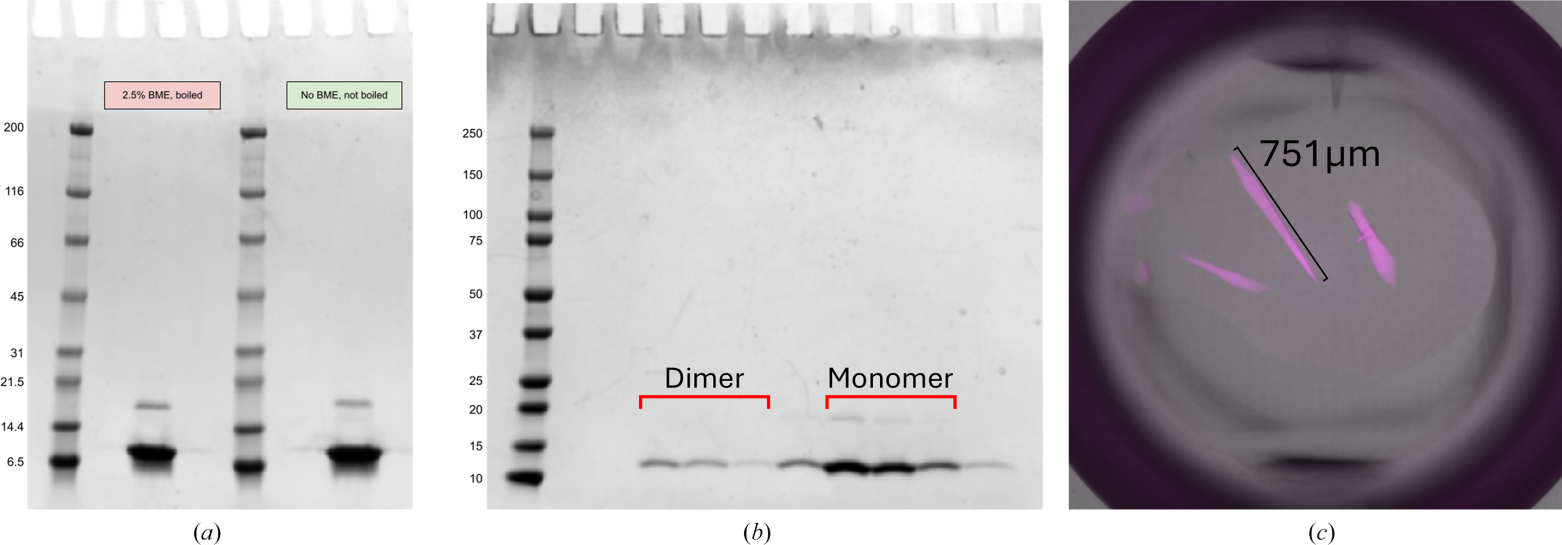
(*a*) SDS–PAGE gel stained with Coomassie Blue showing representative recombinant SR24 used for crystallization under nonreducing and reducing conditions. (*b*) SDS–PAGE gel showing size-exclusion chromatography of SR24. Brackets highlight dimer and monomer peak fractions. Monomer fractions were pooled to obtain the material in (*a*). (*c*) Representative crystal used for X-ray diffraction. UV fluorescence emission indicating protein (magenta) overlaid with bright-field image.

Sequence-verified plasmids were transformed into *E. coli* NiCo21 (DE3) (New England Biolabs) cells and cultured in 1 l LB broth with 100 µg ml^−1^ ampicillin at 37°C. The culture was induced at an OD_600_ of between 0.6 and 0.9 using 1 m*M* isopropyl β-d-1-thiogalactopyranoside (IPTG) and the induced cells were grown for 18 h at 18°C. The cells were harvested by centrifugation and the pellet was then resuspended in lysis buffer (400 m*M* NaCl, 7.8 m*M* KCl, 10 m*M* Na_2_HPO_4_, 1.8 m*M* KH_2_PO_4_ pH 7.5, 10 m*M* imidazole). A cOmplete protease-inhibitor tablet, Pefabloc-SC and DNase I (Roche) were added per the manufacturer’s specifications to prevent protein degradation and minimize DNA contamination. Lysis was performed via sonication at 4°C using a Branson Sonifier 450 set to 50% duty cycle for 30 s pulse intervals totaling 10 min. The homogenous suspension was then centrifuged at 41 060*g* for 30 min at 4°C. The resulting supernatant was flowed over a lysis buffer-equilibrated Qiagen 5 ml Ni–NTA Superflow cartridge followed by 100 ml wash buffer (400 m*M* NaCl, 7.8 m*M* KCl, 10 m*M* Na_2_HPO_4_, 1.8 m*M* KH_2_PO_4_ pH 7.5, 25 m*M* imidazole) and 30 ml elution buffer (400 m*M* NaCl, 7.8 m*M* KCl, 10 m*M* Na_2_HPO_4_, 1.8 m*M* KH_2_PO_4_ pH 7.5, 300 m*M* imidazole). Elution fractions (3 ml) were evaluated for protein content by mixing 10 µl sample with 100 µl Bradford reagent (Bio-Rad), inspected for relative blue appearance and pooled. Dithiothreitol (DTT) was added to the pooled fractions to a concentration of 1 m*M* and SUMO protease, ULP1, was added to cleave the SUMO tag from the N-terminus of SR24 (1 mole per 100 moles of purified SUMO-SR24 protein). The sample was then dialyzed overnight in dialysis buffer 1 (400 m*M* NaCl, 7.8 m*M* KCl, 10 m*M* Na_2_HPO_4_, 1.8 m*M* KH_2_PO_4_ pH 7.5, 1 m*M* DTT) followed by dialysis into dialysis buffer 2 (400 m*M* NaCl, 7.8 m*M* KCl, 10 m*M* Na_2_HPO_4_, 1.8 m*M* KH_2_PO_4_ pH 7.5) with 50 m*M* imidazole and no DTT for 2 h. The resulting sample was incubated for 30 min with HisPure Ni–NTA resin (Thermo Fisher) to bind the cleaved 6×His-SUMO tag, and centrifuged in spin columns to remove the resin, SUMO tag and ULP1. The flowthrough was collected and purified further using a Superdex 75 10/300 GL Increase (Cytiva) size-exclusion chromatography (SEC) column while also undergoing buffer exchange into 20 m*M* HEPES, 200 m*M* NaCl, 1 m*M* TCEP pH 7.5. Fractions containing the 13 kDa target protein were pooled (Fig. 1 ▸), concentrated to 8.12 mg ml^−1^ using a spin concentrator (Amicon Ultra-0.5 Centrifugal Filter Unit, 3 kDa molecular-weight cutoff) and then used for crystallization studies.

### 2.3. Crystallization

The 8.12 mg ml^−1^ sample was subjected to crystallization screening over a broad range of common conditions. Crystals formed in many wells within three days of being set up. Crystals formed the fastest in 0.1 *M* sodium citrate tribasic dihydrate pH 5.6, 0.5 *M* ammonium sulfate, 1 *M* lithium sulfate monohydrate. We harvested representative crystals directly without extra cryoprotectant and shipped them to National Synchrotron Light Source II (NSLSII) for data collection. The conditions for the crystallization of SR24 are summarized in Table 2 ▸.

### 2.4. Data processing, refinement and analysis

The X-ray diffraction dataset used for refinement is summarized in Table 3 ▸. Data were acquired under cryo-conditions on beamline 17-ID-1 at NSLSII, Brookhaven National Laboratory with a wavelength of 0.920085 Å using a Dectris EIGER X 9M detector. We performed initial data processing and error modeling using *HKL*-2000 (Otwinowski & Minor, 1997 ▸), cutting off the resolution at 2.14 Å with a CC_1/2_ of 0.581. The crystals diffracted anisotropically to a high-resolution limit of 2.14 Å with an effective resolution *d*_eff_ of 2.46 Å. The effective resolution was calculated according to the metrics described by Weiss (2001 ▸) and using *EFRESOL* (Urzhumtseva & Urzhumtsev, 2015 ▸). To account for the nonspherical resolution, ellipsoidal truncation and anisotropic scaling was used (Sawaya, 2014 ▸). Resolution limits are noted in Table 3 ▸. Structure solution and refinement was performed using computational resources at the Minnesota Supercomputing Institute (MSI). *AlphaFold*2 (Jumper *et al.*, 2021 ▸) implemented at MSI was used to obtain a molecular-replacement model for the crystallized protein sequence. The structure was solved with *Phaser* (McCoy *et al.*, 2007 ▸) using the *AlphaFold*2-predicted search model and then rebuilt and refined with *Phenix* 1.20.1-4487 (Liebschner *et al.*, 2019 ▸) and *Coot* (Emsley *et al.*, 2010 ▸). *MolProbity* (Chen *et al.*, 2010 ▸) was used for Ramachandran analysis. To confirm that the crystal was not twinned, the data were processed at a lower symmetry and then analyzed with *xtriage* in *Phenix*. The distribution of |*L*| values of the diffraction indicates a twin fraction of 0.00, indicating that the crystal is not twinned. Structure-solution and refinement statistics are listed in Table 3 ▸. The final model was deposited in the Research Collaboratory for Structural Bioinformatics Protein Data Bank as PDB entry 9ec1.

## 3. Results

### 3.1. Crystallization and structure determination

The SR24 crystals (Fig. 1 ▸) belonged to space group *P*6_5_22. The unit-cell parameters were *a* = 106.003, *b* = 106.003, *c* = 614.847 Å, α = 90, β = 90, γ = 120°. There were eight protein molecules in the asymmetric unit. The Matthews coefficient was 4.74 Å^3^ Da^−1^ and the solvent fraction was 74%. Following data acquisition and initial data processing, *AlphaFold*2 was used to generate a model spanning dystrophin residues 2933–3046. The *AlphaFold* model was then used for molecular replacement, yielding a TFZ score of 57.4 and an LLG score of 10861.54. Initial refinement gave an *R*_work_ of 0.2356 and an *R*_free_ of 0.2734. The final *R*_work_ and *R*_free_ values were 0.219 and 0.255, respectively.

### 3.2. Structure analysis

#### 3.2.1. Homology to related spectrin-like repeats

**Figure 2:**
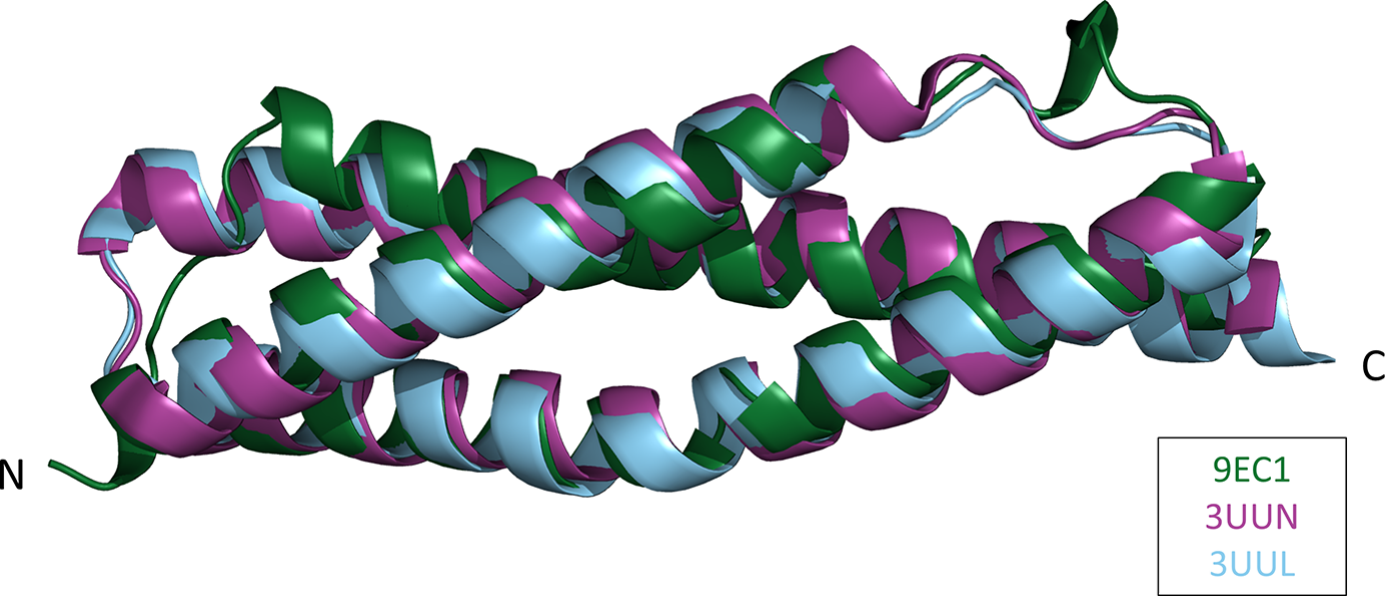
Backbone homology. Alignment of PDB entry 9ec1 (green) chain *A* with PDB entry 3uun (purple; human dystrophin SR1) and PDB entry 3uul (blue; rat utrophin SR1).

The individual chains in the asymmetric unit exhibited high structural similarity, with an all-atom r.m.s.d. average of 1.18 Å and a peptide backbone C^α^ r.m.s.d. of 0.39 Å when compared with chain *A*. The chains exhibit the typical three-helix bundle, helix–turn–helix–turn–helix motif observed in all spectrin-like repeat structures, including spectrin-like repeat 1 of human dystrophin (PDB entry 3uun; Muthu *et al.*, 2012 ▸) and rat utrophin (PDB entry 3uul; Muthu *et al.*, 2012 ▸). SR24 aligns well with both PDB entries 3uun and 3uul, with an r.m.s.d. of 1.137 and 1.18 Å, respectively. The aligned backbones are displayed in Fig. 2 ▸. The structural similarity of SR24 to PDB entries 3uun and 3uul extends to spectrin-like repeat domains found in related cytoskeletal proteins (Table 4 ▸), further supporting the conservation of the spectrin-like repeat fold despite considerable primary-sequence differences.

#### 3.2.2. Intra-chain interactions

**Figure 3:**
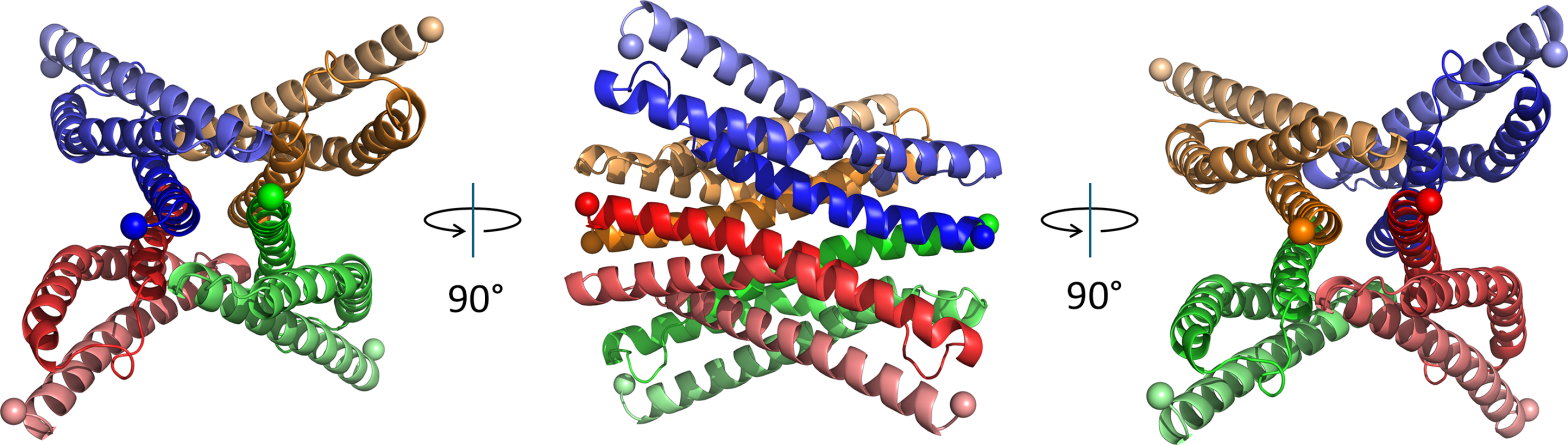
One of two tetramers from the asymmetric unit of PDB entry 9ec1. The chains are differentiated by color. The N- and C-terminus of each chain is highlighted by the first and last C^α^ atom being displayed as a sphere and the progression from the N-terminus to C- terminus is represented by declining color saturation.

The crystal asymmetric unit contains two identical tetrameric assemblies oriented at 90.5° relative to each other. The individual chains in the tetramers alternate in the orientation of the N- and C-termini. The N-termini of each chain form contacts with a parallel chain directly opposite in the tetramer. The interface area of the tetramer composed of chains *A*, *D*, *E* and *H* is 5802 Å^2^ and the area for chains *B*, *C*, *F* and *G* is 5869 Å^2^. The contact area is largely between the faces of helix A (Fig. 3 ▸) and the loop separating helices B and C (sequence IQLSPYN). *AlphaFold*3.0 was used to model a tetramer of SR24. The predicted four-chain SR24 structure is surprisingly similar to the tetramers in the asymmetric unit of PDB entry 9ec1. Aligning the two models results in an r.m.s.d. of 3.63 Å.

The protein was subjected to size-exclusion chromatography prior to crystallization. The chromatogram showed two elution peaks corresponding to monomeric and a higher molecular-weight species. Both species exhibited the same molecular weight by SDS–PAGE, as shown in Fig. 1 ▸. The higher molecular-weight peak comprises 14% of the total protein eluted from the column. Interactions between isolated spectrin-like repeats from dystrophin have been observed before (Calvert *et al.*, 1996 ▸). The physiological relevance of these species is not known as dystrophin is thought to function as a monomer and to not form dimers or tetramers when bound to the dystrophin–glycoprotein complex in muscle (Rybakova & Ervasti, 1997 ▸). We hypothesize that higher molecular-weight interactions between SR24 molecules may reflect the fact that the isolated domain has been removed from its native context where it is in contact with residues of the preceding spectrin repeat 23. *AlphaFold* models of the SR20–SR24 sequence predict interactions between residues of the helix B–C loop of SR24 and the A–B loop of SR23. In the crystal structure, these B–C loop residues form contacts with each other as well as with the N-terminus of adjacent chains, suggesting that they may be responsible for stabilizing higher molecular-weight species in solution.

#### 3.2.3. *B* factors

**Figure 4:**
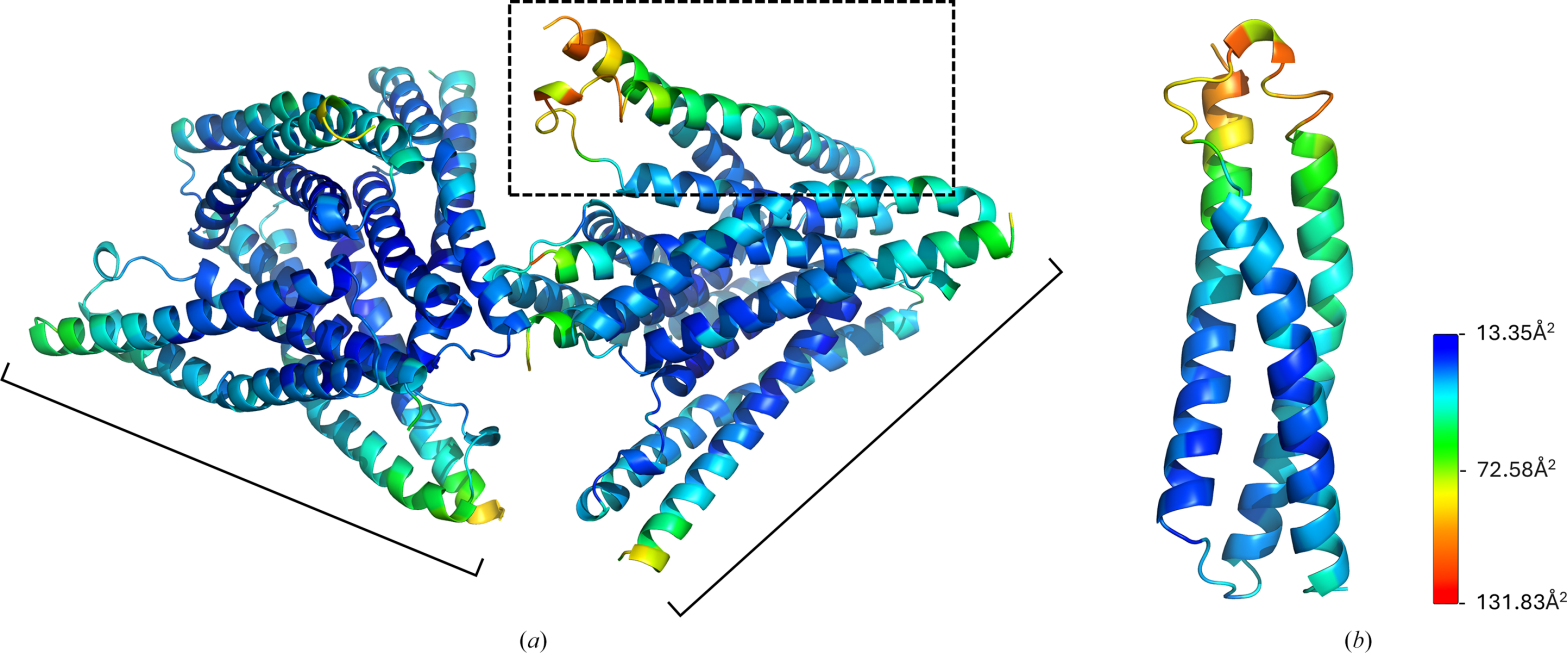
(*a*) *B* factors of the refined model PDB entry 9ec1. The *B*-factor value is represented by color from low (cyan) to high (red). The two tetramers are bracketed, and chain *H* is boxed in. (*b*) Chain *H* of PDB entry 9ec1, the chain exhibiting the highest *B* factors.

The density at the C-terminal end of helix A and the loop region between helices A and B in chain *H* was poor, likely owing to the flexible nature of the loops. The highest *B* factors, except at the N- or C-terminus of each chain, were observed in the A–B loop region. Chain *H* residues 33–48 have an average *B* factor of 88.6 Å^2^, while the average *B* factor of the entire model is 41.9 Å^2^. Overall *B* factors of the eight-chain asymmetric unit are shown in Fig. 4 ▸.

#### 3.2.4. Hydrophobic interactions

**Figure 5:**
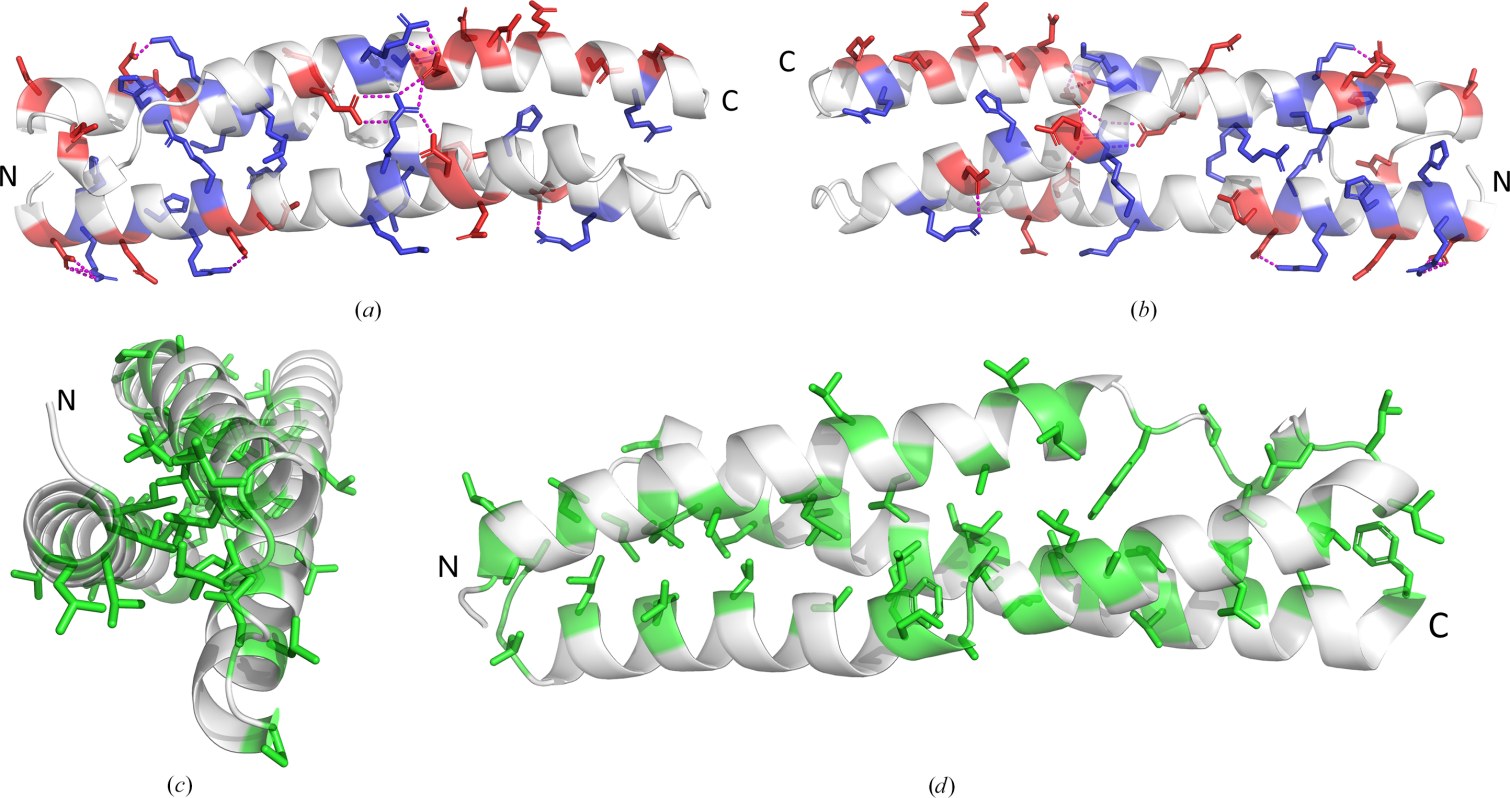
Key interactions that stabilize spectrin-like repeat domains. (*a*, *b*) PDB entry 9ec1 shown from two orthogonal viewpoints with positively (blue) and negatively (red) charged residues colored to show their distribution. Predicted salt bridges are shown by dashed magenta lines. (*c*, *d*) Two viewpoints showing the distribution of hydrophobic residues (green). (*c*) Viewpoint down the central axis of PDB entry 9ec1 showing the packing of hydrophobic residues within the core of the structure. (*d*) Viewpoint along the repeat axis of PDB entry 9ec1.

**Figure 6:**
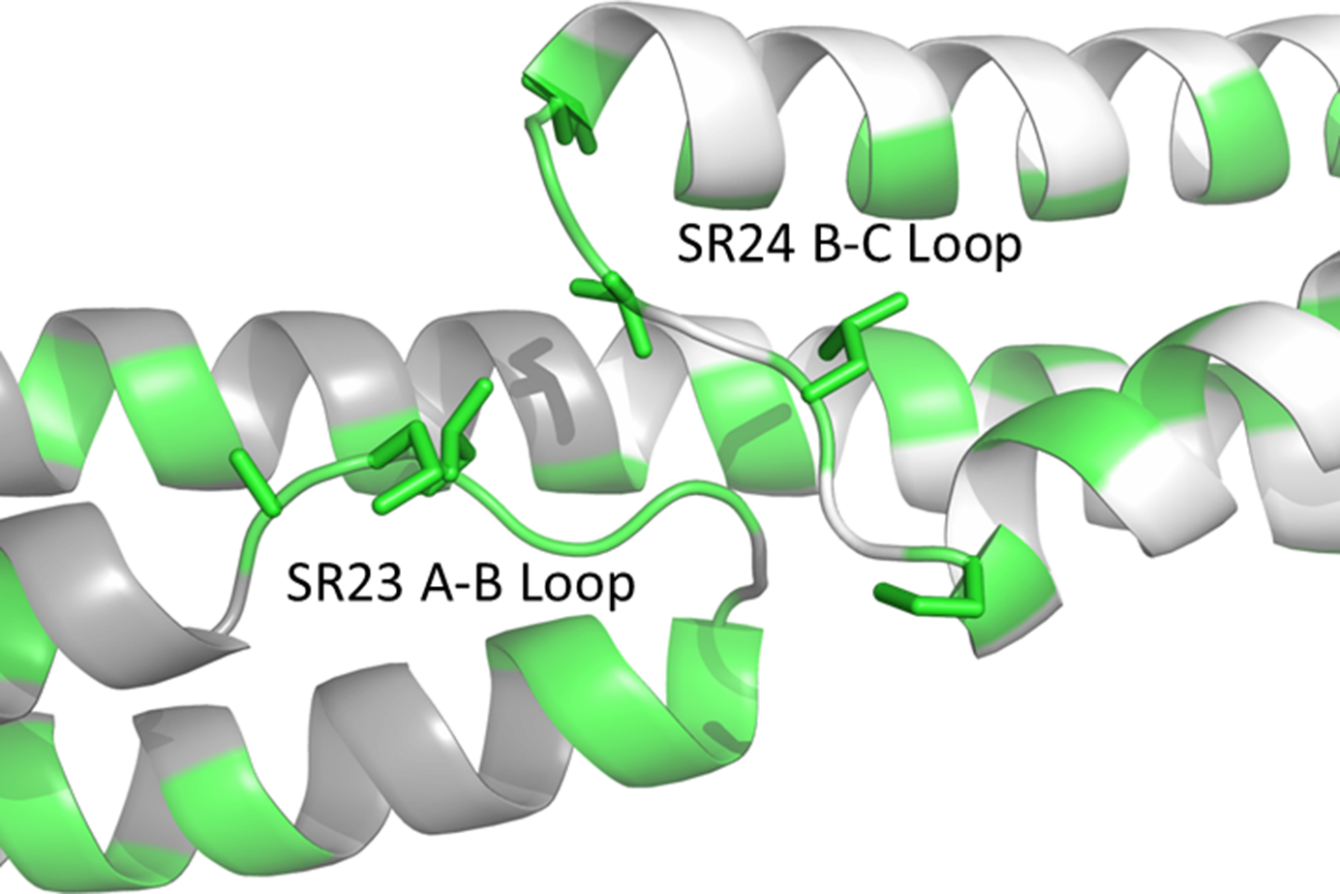
*AlphaFold*-predicted model of the sequence spanning human dystrophin spectrin-like repeats 23 and 24. Hydrophobic residues are colored in green and the side chains of those in the loop region are shown.

The structure reveals molecular details of the interactions that stabilize SR24. The three-helix bundle contains hydrophobic interactions in a heptad repeat common to coiled-coil and other helix bundles with the hydrophobic residues in the core of the helices (shown in Fig. 5 ▸). For example, SR24 and SR1 (PDB entry 3uun) were calculated to have similar buried hydrophobic surfaces areas, with that of SR24 being 6113 Å^2^ and that of SR1 being 6189 Å^2^. Hydrophobic contacts also make up the B–C loop and are a large component of the interaction surface between adjacent chains. In spectrin-repeat proteins, these loops are hypothesized to form contacts between adjacent repeats and in doing so provide a mechanism for structural allostery. *AlphaFold* models of dystrophin SR23–SR24 show the SR24 B–C loop is predicted to form hydrophobic contacts with A–B in SR23 (Fig. 6 ▸).

#### 3.2.5. Salt bridges

**Figure 7:**
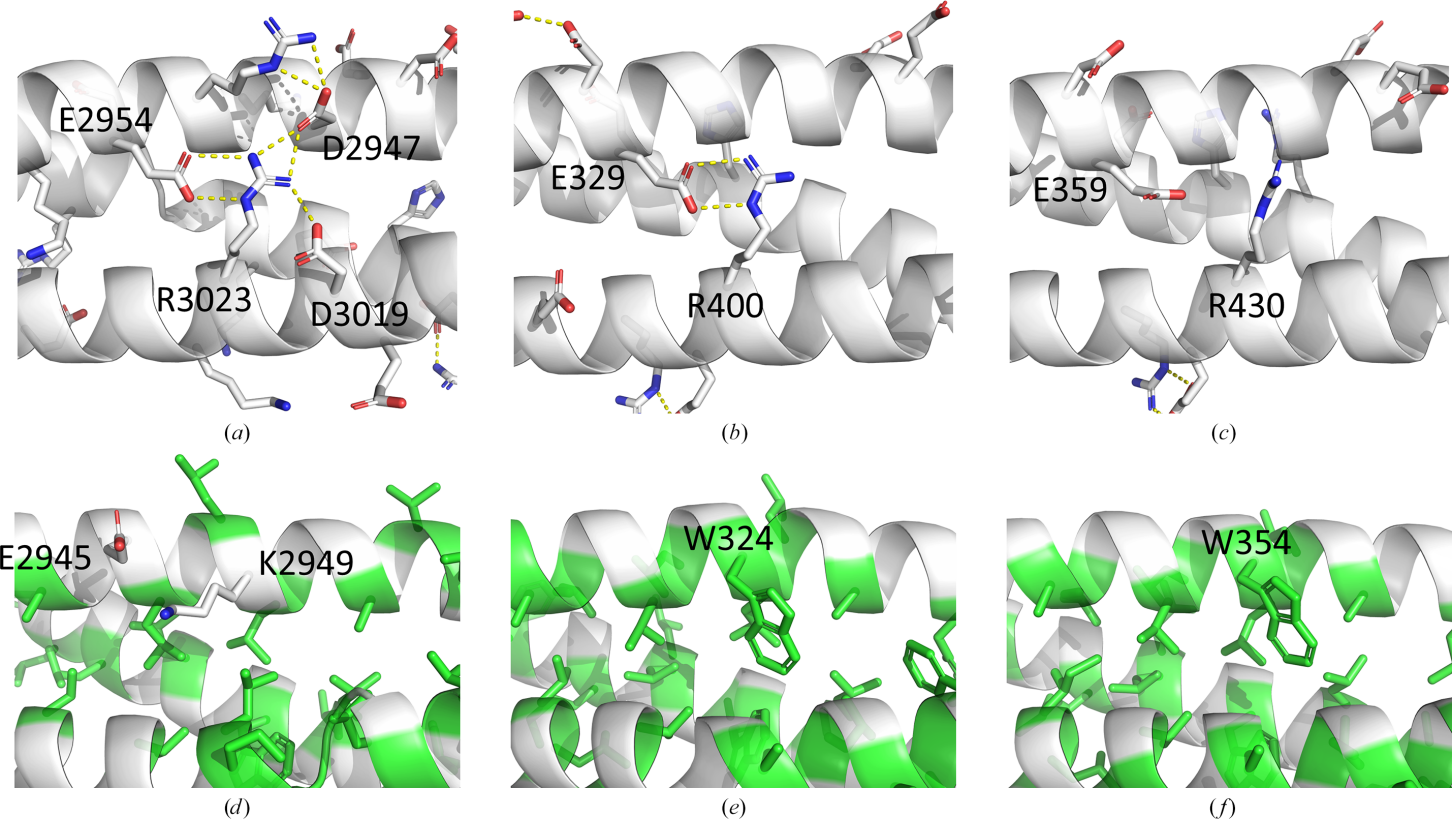
Conserved and nonconserved interactions in PDB entries 9ec1 (*a*, *d*), 3uul (*b*, *e*) and 3uun (*c*, *f*). (*a*)–(*c*) show conserved residues which are predicted to form salt bridges in PDB entries 9ec1 and 3uul. (Salt bridges are represented by yellow dashed lines. Red atoms are oxygen and blue atoms are nitrogen.) (*d*)–(*f*) show the position usually occupied by a conserved tryptophan that clusters with other hydrophobic residues (green), as observed in PDB entries 3uul and 3uun. In PDB entry 9ec1, the same position is instead occupied by a lysine residue (blue) that could interact with a nearby glutamic acid (red) within the same helix.

The SR24 structure reveals a number of inter-helix and intra-helix salt-bridge interactions between the side chains of acidic and basic amino acids. For example, Arg3023 is predicted to interact with Asp2947, Glu2954 and Asp3019. There is also Lys2949 in helix A that exhibits an intra-helix interaction. In most other spectrin-like repeats, a tryptophan involved in hydrophobic packing occupies the same position in helix A. Fig. 7 ▸ compares the interactions in PDB entry 9ec1 with those in the dystrophin and utrophin SR1 crystal structures PDB entries 3uul and 3uun. The key intramolecular interactions observed in dystrophin SR24 (PDB entry 9ec1), SR1 (PDB entry 3uun) and utrophin SR1 (PDB entry 3uul) are summarized in Table 5 ▸.

## 4. Discussion

The 24 spectrin-like repeats of dystrophin form a direct mechanical linkage between the dystroglycan complex (DGC) and cortical actin filaments. The mechanical linkage between the DGC and actin is crucial for sarcolemma integrity and is compromised in Duchenne muscular dystrophy (DMD: Ervasti & Campbell, 1993 ▸; Rybakova *et al.*, 2000 ▸). Investigating the structure of spectrin-like repeats in dystrophin provides molecular insight into their stability, which is important for understanding the native function of dystrophin and how the protein contributes to the mechanical properties of the sarcolemma. It also provides support and reference for designing optimized dystrophin replacements to treat DMD that utilize an engineered dystrophin surrogate protein that contains, out of necessity, non-native spectrin-like repeat domain boundaries (Mendell *et al.*, 2023 ▸; Roberts *et al.*, 2023 ▸; Zaidman *et al.*, 2023 ▸). Importantly, spectrin repeat 24 is a conserved component of all dystrophin-replacement therapies (Roberts *et al.*, 2023 ▸) and has been shown to be required for rescuing disease phenotypes in animal models (McCourt *et al.*, 2015 ▸; Ramos *et al.*, 2019 ▸). The isolated SR24 domain is one of the most stable domains in dystrophin: published studies show that it exhibits a thermal denaturation temperature of 64.3°C. Neighboring domains exhibit similarly high thermal stability, as does a C-terminal fragment consisting of SR18-CT, which exhibits a denaturation transition at 65.5°C. Conversely, the isolated N-terminal SR1 domain denatures at 54.1°C, while an N-terminal fragment containing SR1 (NT-R10) is even less stable, unfolding at 46.1°C (Henderson *et al.*, 2011 ▸; Mirza *et al.*, 2010 ▸).

The thermal stability of SR24 reflects the atomic inter­actions that stabilize the folded state, including hydrophobic interactions within the three-helix bundle core of the repeat as well as salt-bridge interactions between and along helices. Interestingly, the structure of SR24 shows salt bridges that are present in SR24 but not in SR1. The salt bridges likely help to stabilize SR24, contributing to the overall stability of the C-terminal domains of dystrophin. The resolution of additional stabilizing interactions in SR24, not present in SR1, provides a roadmap for modifying the stability of dystrophin-replacement therapies and for evaluating the potential stability of exon-skipped dystrophins that contain non-native hybrid repeats with bundled helices that do not normally interact and may, as a result, have fewer stabilizing inter­actions (Menhart, 2006 ▸).

**Figure 8:**
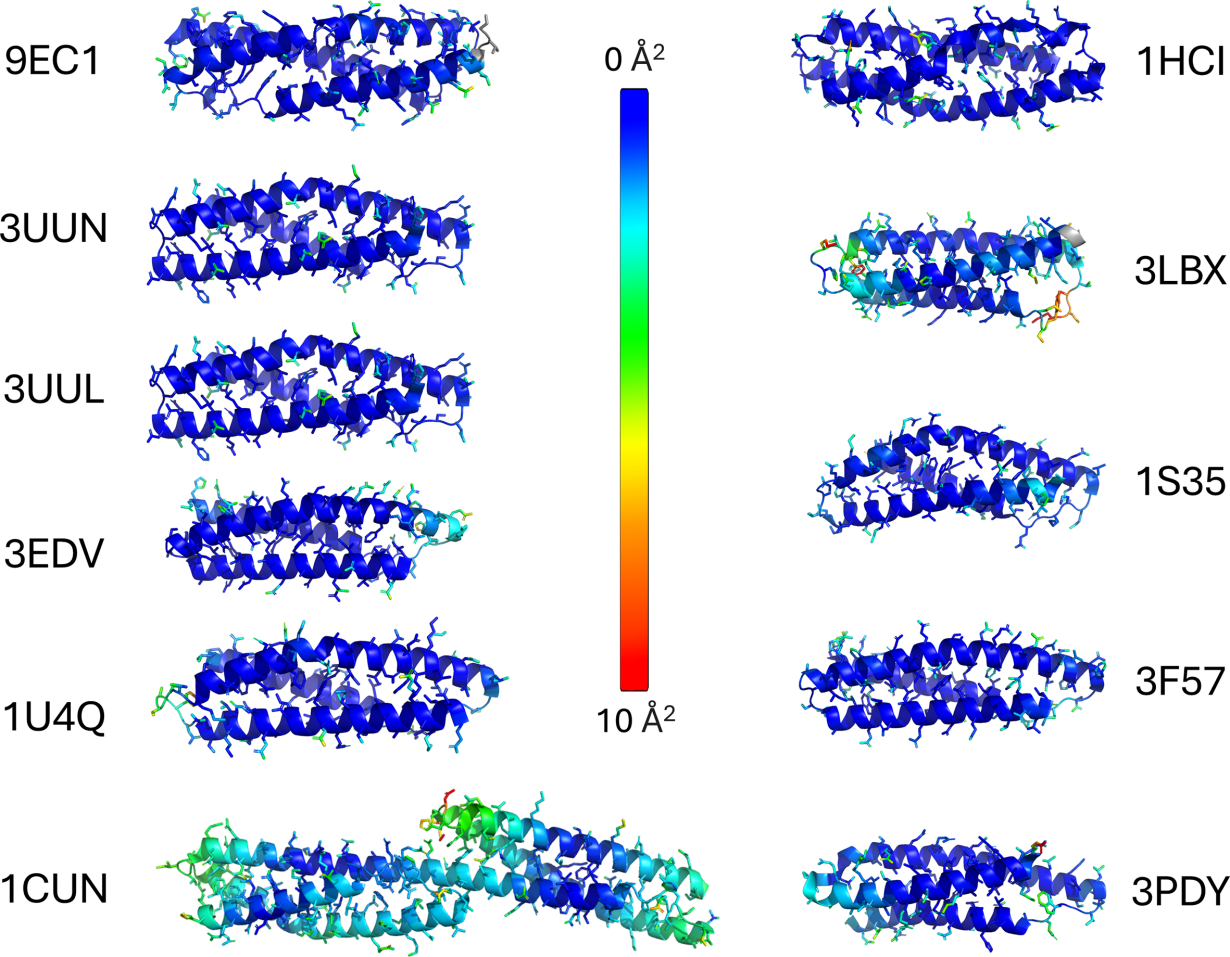
Alignment of *AlphaFold* models with existing spectrin-like repeat crystal structure models. The r.m.s.d. for each atom is represented by color, from low (cyan) to high (red)

Spectrin-like repeats 1 and 24 are the only two repeats in dystrophin that have been solved by X-ray crystallography thus far. As we solved the structure of SR24, we found models of the repeat predicted by *AlphaFold* were highly comparable to the experimentally obtained SR24 structure PDB entry 9ec1 (Fig. 8 ▸). The single-chain all-atom r.m.s.d. between chain *A* in PDB entry 9ec1 and the *AlphaFold* model of SR24 is 1.13 Å and the backbone r.m.s.d. is 0.53 Å. Similarly, the *AlphaFold* prediction of SR1 aligns with the solved SR1 structure (PDB entry 3uun) with an all-atom r.m.s.d. of 1.47 Å and a backbone r.m.s.d. of 0.79 Å. Importantly, SR24 was not included in the data used to train *AlphaFold*. *AlphaFold* predicts the structures of other spectrin-like repeat proteins to a similar degree of accuracy compared with existing crystal structures. All-atom r.m.s.d.s for these comparisons are summarized in Table 4 ▸. The accuracy of *AlphaFold* in predicting spectrin-like repeats supports predicted structures of the rest of the entire dystrophin rod domain, comprised of 24 spectrin repeats. *AlphaFold* models of the dystrophin rod domain offer insight into the design and testing of next-generation dystrophin-replacement therapies, including existing and next-generation micro-dystrophins (Bengtsson *et al.*, 2025 ▸), split intein self-splicing full-length dystrophins (Tasfaout *et al.*, 2024 ▸) and exon-skipping therapies that result in the expression of dystrophin surrogates with non-native spectrin-like repeats (Roberts *et al.*, 2023 ▸).

The accuracy of *AlphaFold*-predicted spectrin-like repeat models suggests that they will also be a useful starting point for determining the exact boundaries of spectrin-like repeat domains in dystrophin and homologous proteins. The current proposed boundaries are based on sequence homology (Winder *et al.*, 1995 ▸), but have not been extensively tested experimentally. Our SR24 structure provides an example of the use of *AlphaFold* to determine domain boundaries. The *AlphaFold* prediction, which informed our construct design, identified six additional residues C-terminal to SR24, in what is typically annotated as Hinge 4, that were predicted to form a stable helix at the C-terminus of SR24. We included these residues in the SR24 protein used for crystallization. The PDB entry 9ec1 SR24 structure shows that the Hinge 4 residues (amino-acid sequence AHRDFG) form a helix that interacts with the A–B loop of SR24, thus demonstrating the utility of using *AlphaFold* structural models to define spectrin-repeat boundaries when designing dystrophin-replacement strategies, rather than relying solely on sequence homology-derived domain boundaries.

## Supplementary Material

PDB reference: dystrophin spectrin-like repeat 24, 9ec1

## Figures and Tables

**Table 1:** Macromolecule-production information

Source organism	Human
DNA source	Integrated DNA Technologies
Expression vector	pTD68
Plasmid-construction method	Gibson assembly
Expression host	*E. coli* BL21
Complete amino-acid sequence	STGLERLQELQEATDELDLKLRQAEVIKGSWQPVGDLLIDSLQDHLEKVKALRGEIAPLKENVSHVNDLARQLTTLGIQLSPYNLSTLEDLNTRWKLLQVAVEDRVRQLHEAHRDFG

**Table 2:** Crystallization

Method	Sitting-drop vapor diffusion
Plate type	Low-profile INTELLI-PLATE 96-3
Temperature (K)	295
Protein concentration (mg ml^−1^)	8.12
Buffer composition of protein solution	20 m*M* HEPES pH 7.5, 200 m*M* NaCl, 1 m*M* TCEP
Composition of reservoir solution	0.1 *M* sodium citrate tribasic dihydrate pH 5.6, 0.5 *M* ammonium sulfate, 1 *M* lithium sulfate monohydrate
Volume and ratio of drop	100 nl, 1:1 ratio
Composition of cryoprotectant	Direct cooling, no extra cryoprotectant added
Drop setting	ARI Phoenix
Seeding	No

**Table 3:** Data-collection and refinement statistics

Data collection	
X-ray source	17-ID-1, NSLSII
Wavelength (Å)	0.920085
Detector	Dectris EIGER X 9M
Exposure time (s)	0.02
Crystal-to-detector distance (mm)	300
Angle increment (°)	0.2
Resolution range (Å)	34.48–2.141 (2.217–2.141)
Effective resolution range (Å)	34.48–2.46 (2.51–2.46)
Resolution limits in each direction (Å)	
0.894*a* − 0.447*b*	2.741
*b*	2.741
*c*	2.026
Space group	*P*6_5_22
*a*, *b*, *c* (Å)	106.00, 106.00, 614.85
α, β, γ (°)	90, 90, 120
Matthews coefficient (Å^3^ Da^−1^)	4.735
Solvent content (%)	74
Total reflections	1360479
Unique reflections	71449 (551)
Multiplicity	19.0
Mosaicity (°)	0.2
Completeness (%)	62.53 (4.93)
Completeness at effective resolution (%)	100 (100)
Mean *I*/σ(*I*)	13.1
Wilson *B* factor (Å^2^)	33.6
*R*_merge_	0.196 (1.643)
*R*_meas_	0.202 (1.704)
*R*_p.i.m._	0.046 (0.448)
CC_1/2_	0.999 (0.581)
Refinement statistics	
Reflections used in refinement	71437 (551)
Reflections used for *R*_free_	3592 (25)
*R*_work_	0.2147 (0.3631)
*R*_free_	0.2544 (0.4892)
No. of non-H atoms	
Total	7630
Macromolecules	7455
Ligands	75
Solvent	100
No. of protein residues	929
R.m.s.d., bond lengths (Å)	0.005
R.m.s.d., angles (°)	0.67
Ramachandran favored (%)	98.25
Ramachandran allowed (%)	1.42
Ramachandran outliers (%)	0.33
Rotamer outliers (%)	1.58
Clashscore	3.98

**Table 4:** Structural alignment of PDB entry 9ec1 and related proteins

		Comparison with PDB entry 9ec1			All-atom r.m.s.d. to *AlphaFold* (Å)		
Protein name	PDB code, chain	Identical residues	Sequence identity (%)	R.m.s.d. (Å)	Overall	Helices	Loops
Human dystrophin spectrin-like repeat 24	9ec1, *A*	—	—	—	1.13	1.12	1.07
Human dystrophin spectrin-like repeat 1	3uun, *A*	25	21.9	1.37	1.49	1.25	1.32
Rat utrophin spectrin-like repeat 1	3uul, *A*	25	23.1	1.18	1.03	0.98	0.80
Chicken brain β-spectrin repeat 14	3edv, *A*	28	26.4	2.45	1.42	1.37	1.73
Chicken brain α-spectrin-like repeat 15	1u4q, *A*	24	21.8	1.45	1.38	2.48	13.61
Chicken brain α-spectrin-like repeat 16	1cun, *A*	23	20.7	1.48	1.41	2.09	1.22
Chicken brain α-spectrin-like repeat 17	1cun, *A*	25	22.1	2.25	1.49	1.22	2.24
Human skeletal muscle α-actinin-2 repeat 2	1hci, *A*	15	13.9	2.20	1.25	1.16	1.48
Human erythrocyte α-spectrin-like repeat 1	3lbx, *A*	22	19.0	2.63	2.87	2.50	15.10
Human erythrocyte β-spectrin-like repeat 8	1s35, *A*	27	21.6	2.79	1.24	1.08	0.70
Human erythrocyte β-spectrin-like repeat 14	3f57, *A*	25	22.5	2.57	1.18	1.06	1.38
Human plectin repeat 3	3pdy, *A*	19	18.1	3.18	1.58	1.32	1.23

**Table 5:** Comparison of intramolecular interactions of dystrophin and utrophin spectrin-like repeats calculated using *Maestro* (Schrödinger, release 2025-3) following the addition of atoms missing from the protein chain, protein preparation and protonation at pH 7.4 and energy minimization

Protein name	PDB code, chain	Total hydrogen bonds	Salt bridges	Aromatic hydrogen bonds	π–π stacking	π–cation bonds	% helix	% loop	Molecular charge	Buried hydrophobic surface area (Å^2^)
Human dystrophin SR24	9ec1, *A*	164	12	2	0	2	77.9	22.2	−6	6113
Human dystrophin SR1	3uun, *A*	151	6	5	2	0	87.0	13.0	−21	6189
Rat utrophin SR1	3uul, *A*	160	6	7	2	0	89.7	10.3	−12	6432
